# Healthcare Workers’ Resilience Toolkit for Disaster Management and Climate Change Adaptation

**DOI:** 10.3390/ijerph191912440

**Published:** 2022-09-29

**Authors:** Heba Mohtady Ali, Jamie Ranse, Anne Roiko, Cheryl Desha

**Affiliations:** 1 Cities Research Institute, Griffith University, Gold Coast, QLD 4215, Australia; 2 School of Engineering and Built Environment, Griffith University, Gold Coast, QLD 4215, Australia; 3 Department of Emergency Medicine, Griffith University, Gold Coast, QLD 4215, Australia; 4 Menzies Health Institute Queensland, Griffith University, Gold Coast, QLD 4215, Australia

**Keywords:** healthcare workers (HCWs), hospitals, climate change adaptation, disaster resilience, toolkit, management

## Abstract

Climate change has been recognised as a multiplier of risk factors affecting public health. Disruptions caused by natural disasters and other climate-driven impacts are placing increasing demands on healthcare systems. These, in turn, impact the wellness and performance of healthcare workers (HCWs) and hinder the accessibility, functionality and safety of healthcare systems. This study explored factors influencing HCWs’ disaster management capabilities with the aim of improving their resilience and adaptive capacity in the face of climate change. In-depth, semi-structured interviews were conducted with thirteen HCWs who dealt with disasters within two hospitals in Queensland, Australia. Analysis of the results identified two significant themes, HCWs’ disaster education and HCWs’ wellness and needs. The latter comprised five subthemes: HCWs’ fear and vulnerability, doubts and uncertainty, competing priorities, resilience and adaptation, and needs assessment. This study developed an ‘HCWs Resilience Toolkit’, which encourages mindfulness amongst leaders, managers and policymakers about supporting four priority HCWs’ needs: ‘Wellness’, ‘Education’, ‘Resources’ and ‘Communication’. The authors focused on the ‘Education’ component to detail recommended training for each of the pre-disaster, mid-disaster and post-disaster phases. The authors conclude the significance of the toolkit, which provides a timely contribution to the healthcare sector amidst ongoing adversity.

## 1. Introduction

Climate change is recognized as a critical element of public health and signifies one of the most critical threats affecting global health [1,2]. Combatting climate change presents the greatest global health opportunity of the 21st century. Natural disasters and pandemics caused by emerging microbial pathogens have been witnessed worldwide and have focused greater attention on the complex relationships between our environment, our changing climate, and our health [3,4]. In the meantime, prioritising appropriate climate change mitigation and adaptation actions will improve health outcomes and avert disaster-related disturbances in health care delivery [5]. Specifically, enhanced planning, monitoring and assessment approaches are required to build the resilience of hospitals in the face of disasters, and other climate change impacts [6,7].

HCWs are suffering from climate change impacts on population health and healthcare services [8]. Hospitals are unique in having a wide range of healthcare workers constituting the hospitals’ human resources. HCWs are entirely responsible for health care delivery and patients’ safety, directly by doctors, nurses, and allied health professionals, and indirectly by medical assistants, technicians, and waste handlers [9,10,11]. HCWs are universally influenced by work-related stress in various healthcare settings [12,13,14]. Such stress is magnified during disasters (HCWs), owing to the increased workload, multitasks, roles and time constraints [15,16]. Moreover, the physical stress and emotional exhaustion faced by HCWs’ during disasters are exacerbated by having to deal with traumatic situations while experiencing their own fear, uncertainty, and concerns regarding the impact of such disasters on their homes, and families [17,18,19,20,21,22]. For example, nurses demonstrated exaggerated liability for Post-Traumatic Stress Disease, major depression, or severe psychological illness during disasters [20,23]. Equally important COVID-19 pandemic has resulted in the death of many HCWs and augmented their psychological stress and alcohol consumption [13,24,25,26,27].

Resilience has both physical and social dimensions in healthcare settings. A hospital’s resilience is defined as its ability to survive, absorb, acclimatise, adapt to, change and recover from the hazard effects in a timely and efficient manner [28]. It follows that resilience metrics used to capture a hospital’s performance during disasters then, would consider various factors in the hospitals’ performance during disasters as the physical factor, including “structural” and “non-structural” components, and the social “functional” one [29]. Human resources, communication and information management are crucial components of the latter [29,30,31,32,33]. Hence, regardless of physical resilience, human behaviour as HCWs’ resilience, preparedness, training, education, practice, and safety contribute to the hospital’s resilience during the disaster [16,34,35].

HCWs do not have a sufficient disaster preparedness and adequate competencies to ensure their safety and excel in their practices while facing the dreadful impacts of current and future disasters [7,36]. In addition, scarce experimental information was reported on HCWs’ resilience-enhancing strategies [17]. Considering that resilience implies learning from past failures and successes [37,38]. Thus, research should focus on HCWs’ resilience and explore their relevant capabilities, practices, safety, preparedness, and learning [7].

This paper considers the local context of disaster response in Australia. It explores how hospital managers and decision-makers can enhance their healthcare workers’ capabilities to improve business continuity and build personal and organisational resilience to future crises and climate change impacts. The researchers addressed the following research question: How can hospital managers and decision makers improve HCWs’ disaster resilience?

## 2. Methods

### 2.1. Design

A qualitative case-study design was adopted, including in-depth and semi-structured interviews. Interviews were designed to enable participants to reflect on what happened, anticipated, and responded to disasters [39,40]. Their perceptions were sought about issues related to staff awareness, training, education, and other capacity-building insights gained through experiences in dealing with disasters as HCWs.

### 2.2. Participant Recruitment

One hospital is located in the Gold Coast and the other in Brisbane, both very densely populated parts of Southeast Queensland, Australia. These hospitals were selected to ensure that both were exposed to similar challenges, had comparable opportunities, hierarchies and political regime management structures and followed the same policies.. Throughout this article, we will use (H1) and (H2) to report on these two hospitals.

The research team liaised with two hospital advisors (one for each hospital) to discuss the recruitment of participants, and to ensure the questions and logistics for the study were appropriate for the hospital workers.

The hospital advisor in one hospital was the disaster and emergency management coordinator, and in the other hospital was a Nurse Manager (Nursing Executive). The responsibility of each of the advisors was being the chief point of contact for the research team’s activities with their hospitals. The sampling methods were limited to the services and departments approved for the project by liaising with the hospitals’ management.

The two advisors then distributed information about the study to potential hospital staff participants via email, including managers who had experience in working through disasters (e.g., bushfires, floods, COVID-19, locally significant incidents). Once potential interviewees indicated their interest to the advisors, the researchers followed up with a meeting invitation email. Interviews were arranged to be face-to-face or virtual (using Microsoft Teams), based on the preference of the interviewee.

### 2.3. Data Collection and Analysis

The interviews were conducted confidentially by two researchers (the first author was the leader, and one of the co-authors was an observer) between June 2021 and April 2022. Each interview lasted approximately 45–60 min.

With each participant’s permission, their interview was audio-recorded using the recording function on Microsoft Teams and on the mobile phone of one of the researchers as a backup. Each interview recording was transcribed verbatim by the first author using Microsoft Word and saved in a file format compatible with NVivo. Participants’ details, interview transcripts, and recordings were coded with a participant number for discussion and publication purposes to maintain confidentiality. These were only accessible by the researchers named on the ethics approval.

Thematic analysis was used to analyse the interview transcripts using Braun and Clarke’s framework as a guide [41]. This was supported by the features within the NVivo software, enabling a detailed and nuanced account of the data to be produced. Moreover, reflections based on a synthesis of participants’ voices were constructed.

### 2.4. Quality Assurance

The validity and trustworthiness of the data analysis were addressed by ensuring transparency and consistency in the coding process [42]. An audit trail was provided of how and when any codes were created or renamed and how key themes were identified through coherence and exemplified appropriately. The research team discussed the meanings and interpretations of the codes and themes to minimise biased reporting and identify areas where information is likely to be missing.

## 3. Results

Out of 21 invited hospital staff members, 13 were available to be interviewed. All the participants were over 18 years of age, including 10 females (5 from each hospital) and 3 males (one from H1 and two from H2). The participants performed a range of duties in their workplace roles, including management, consultancy, directorship, and advisory roles.

**Figure 1:**
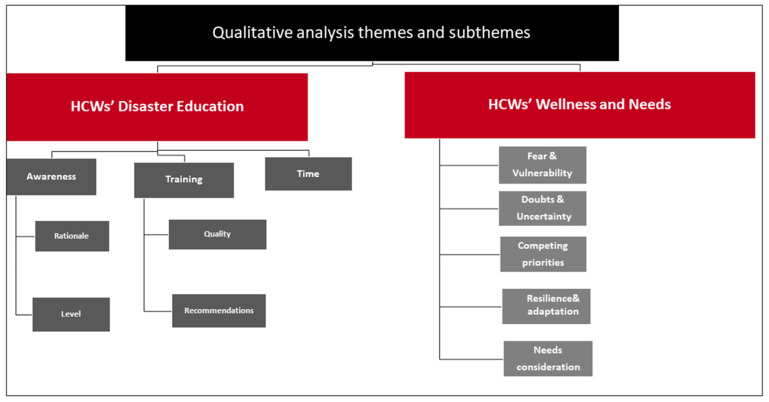
This is a figure showing the adopted themes and subthemes of the analysis of the results.

The current study has two significant themes (HCWs’ disaster education and HCWs’ wellness and needs), eight subthemes and four sub-sub themes. The first theme addressed the participants’ perceptions and descriptions of their disaster planning education, focusing on their awareness, training, and time factor. The second theme explored the participants’ perceptions of wellness and needs during various disasters (Figure 1). The results are presented to include the participants’ transcripts and the authors’ constructed reflections based on synthesised participants’ voices.

### 3.1. HCWs’ Disaster Education Regarding Disaster Planning and Preparedness (DPP)

This theme includes the participants’ perceptions regarding their DPP awareness, training, and time as three subthemes. The first subtheme illustrates the participants’ perceptions of their awareness of disaster planning and preparedness (DPP); including the reasons and methods that led them to be aware of their hospitals’ DPP documents and processes and the knowledge and competencies gained as because of this awareness (See Table 1). The second subtheme depicts the participants’ needs, expectations and thoughts regarding their DPP training and practice quality. It also includes the participants’ recommendations regarding the training and simulation (See Table 2). The third subtheme shows the participants’ perceptions regarding the significance of time and its effect on the quality and application of DPP training (See Table 3). Each table shows excerpts from participants’ transcripts and the constructed reflections based on synthesized participants’ voices.

### 3.2. HCWs’ Wellness and Needs

This theme includes the participants’ perceptions of their wellness and needs. This theme consists of the participants’ perceptions regarding the following subthemes: ‘Fear and Vulnerability’, ‘Doubts and uncertainty, ‘Competing priorities’, ‘Resilience and adaptation’ and ‘Needs considerations’ (See Table 4).

## 4. Discussion

The resilience of a system is its competency to cope with catastrophes or disruptions by reacting in ways that preserve its core function, structure, and identity whereas keeping its ability to adapt, learn, and transform [43]. In addition, the individual’s resilience implies their adaptive responses to stress and their commitment to values and goals regardless of experiencing hardship [44]. The current study addressed how hospital decision-makers can improve HCWs’ resilience to disasters, including education and wellness situations.

### 4.1. Enhancing Education and Training

The participants indicated that DPP awareness might be mandatory for their job tasks, roles, and committees (H1.4 and 6 and H2.4 and 6). However, there were several considerations regarding the quality, effectiveness and mandatory level of such awareness, their gained competencies and the depth of knowledge. The participants had difficulties and challenges in applying what they had been taught in their DDP training. Thus, such activity does not ensure effectiveness and competency (H1.1, 5 and 6 and H2.5 and 6).

In addition, the participants’ recommendations included enhancing the level of DPP training and providing both junior and senior staff with mandatory, annual and sustainable training that helps them learn from past practices and think about various scenarios and possibilities. These recommendations highlighted the need for simulations based on authentic learning experiences and count the human factor during actual life application of lessons learnt. For instance, it should consider preparing the staff to work if electronics fail due to disasters. Monitoring and evaluating HCWs’ education and training should guarantee updated content (H1.1, 2 and 6 and H2.4 and 6).

Similar recommendations were described in the literature regarding the development of HCWs via awareness and training [2,7,38].

Such development should include disaster-specific contents. DPP training can develop HCWs’ knowledge and skills and improve their response to crises and emergencies [45,46].

Previous research studies showed that HCWs’ development should cover other relevant areas necessary to augment staff competencies [8,47,48,49,50,51]. For instance, in case of infectious diseases disasters, HCWs should be well educated and trained regarding proper infection control practices, quarantine processes, and how to treat, isolate, report, and track patients using electronic systems [47,50,51]. In addition, leadership training for hospital management and the development of emergency management operations plans are crucial for all types of disasters [52,53]. Moreover, HCWs should learn more about the climate change impacts on health, the environment and societies, and the climate change mitigation and adaptation strategies and approaches [8,54].

In the present study, the participants indicated that they were struggling with the time limitation. Moreover, it was evident that the HCWs’ limited time hinders their ability to understand the hospital disaster management plan deeply. Some consider education a time-consuming and exhausting process. Hence, time versus effort may be a consideration (H1.3 and 4 and H2.3 and 4). These findings conformed with a research study conducted by Besley, Dudo [55] and illustrated that the HCWs have high levels of commitment to keep training and education about climate change, and its impact on health. Yet, many participants indicated several barriers that obstruct them from pursuing such training. Among these barriers, time restraints were the most identified one [55].

### 4.2. Beyond Education and Training: The HCW’s Call for Action (SOS)

Climate-change–related hazards and adverse events with unfavourable impacts, such as disasters, affect the globally available vulnerable people and lead them to struggle with deep emotional traumas [56,57,58,59]. Such overwhelming situations significantly affect health professionals due to the nature of their routine work that predisposes them to high exposure levels [60].

In the current study, many stressful factors and concerns were raised in exploring the participants’ wellness and needs, including fear about their well-being, family safety, tiredness, and vulnerability. Some expressed uncertainty and doubts regarding their knowledge levels, coping with changes, resilience, and adaptations—moreover, the stress they encountered from competing priorities and time limitations. Several participants in the current study highlighted the significance of considering their needs by providing them with adequate resources, PPE and equipment, respecting their mental and psychological wellness, and most importantly, thinking of them as humans, not only as health professionals, and establishing a “well-being committee” (H1.1, 2, 3 and 5 and H2.2, 4, 5 and 7).

Similar stressful factors were identified in other studies due to lack of resources, lack of clarity in communications, deficient, excessive, rapidly changing information and competing priorities. [47,61]. Hence, during disasters and epidemics, more consideration should be given to the mental health of healthcare workers by enhancing their work experience and providing them with psychological, personal and family support [26].

### 4.3. A Conceptual Framework for Action—A Healthcare Workers’ Resilience Toolkit for Their Disaster Management and Climate Change Adaptation

Globally, several types of disasters, extreme weather, and climate events are expected to increase in frequency and intensity in the coming decades. Hence, these unfavourable situations will majorly impact health care systems and HCWs [56]. Climate change adaptation refers to activities that sustain or augment the adaptive capacity and resilience aiming to reduce the human or natural system’s vulnerabilities to climate change risks and impacts [54].

There are a few scenarios to acknowledge, creating a particular problem space to work into. Each of the training scenarios still requires consideration of the three domains cognitive (mental thinking), affective (attitudes), and psychomotor competencies (utilising motor skills and coordinating them) [62]. In the practitioner’s world, these competencies are perceived as applied practises for performance and change scoring and coordination [63].

Staff practices and safety, communication, equipment and resources were among the nine learning areas crucially highlighted in the Hybrid Resilience Learning Framework (HRLF) for both organisational resilience and learning components by Ali, Ranse [38]. Moreover, the current study conveyed an emergency ‘SOS’ call from the participating interviewees, including the key factors that affect their resilience in the face of disasters.

**Figure 2:**
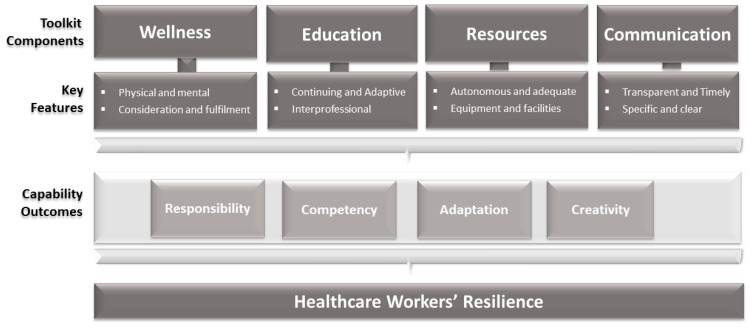
A schematic of the ‘HCWs Resilience Toolkit’, highlighting key factors that can enhance disaster management and climate change adaptation.

Hence, the authors in the current study responded to the HCWs expressed needs and their call for action by proposing an ‘HCWs Resilience Toolkit’, which encourages mindfulness amongst leaders, managers and policymakers about supporting four priority HCWs’ needs: ‘Wellness’, ‘Education’, ‘Resources’ and ‘Communication’. First, this framework shows the value of listening to the HCWs’ voices and concerns. Second, it emphasises the magnitude of having a culture of taking training seriously via mandatory and sustainable authentic inter-professional learning experiences. Third, it focuses on timely, clear, specific, and transparent communication with HCWs. Finally, it underscores providing HCWs with all the required resources and equipment (Figure 2).

**Figure 3:**
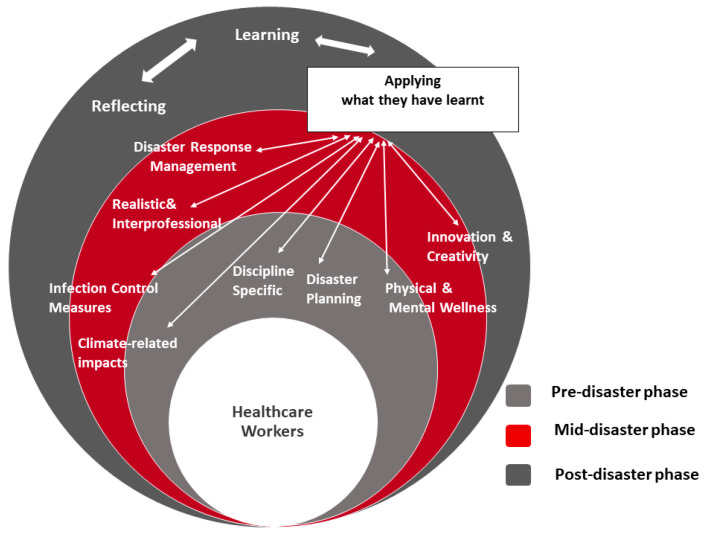
The educational component of the Healthcare Workers’ Resilience Toolkit: education, training, and practice implications (pre-disaster, mid-disaster and post-disaster phases).

The authors focused on the ‘Education’ component of the ‘HCWs Resilience Toolkit’ to detail recommended training for each of the pre-disaster, mid-disaster and post-disaster phases (Figure 3). The suggested topics are not sharply restricted for each phase, as many of them span the three phases, for instance, training related to climate change impacts, infection control measures, physical and mental wellness, inter-professionalism, innovation, and creativity. In post-disasters phases, Hospital managers should provide opportunities for HCWS to reflect in debriefing sessions and learn and apply what they have been taught while facing disasters to boost their performance in future ones.

## 5. Limitations of the Study

Given the topic area of disaster resilience, there was a risk that the researchers may impact upon political or institutional sensitivities regarding classified information, public awareness campaigns, and past events. To minimise this potential, the researchers ensured that political and institutional sensitivities form part of the discussions with the project advisors prior to each round of interviews.

Moreover, there were the limitations of in-depth interviews being not generalisable, time-consuming, requiring a more prolonged verification process, and difficult to add context.

## 6. Conclusions

Hospital managers and decision makers should pay more attention to the HCWs’ awareness, training and wellness. Adopting the proposed ‘‘HCWs Resilience Toolkit’ by hospital leaders, managers and policymakers will potentiate and expand HCWs’ competencies and adaptation and decrease their stress and vulnerability. Moreover, it will help the HCWs be more adaptive, competent, creative, and responsible, hence ensuring their resilience. Such resilient, empowered, and supported HCWs can boost the hospitals’ resilience and business continuity while facing the current and future climate change impacts and diverse disasters. The authors conclude the significance of the toolkit, which provides a timely contribution to the healthcare sector amidst ongoing adversity. There are immediate opportunities for the toolkit to support hospitals in managing disruptions to hospital functions, through enhancing their HCWs’ adaptive capacity, competence, creativity and responsibility.

## Figures and Tables

**Table 1:** The awareness of disaster planning and preparedness (DPP): This is a table that shows excerpts from participants’ transcripts and the constructed reflections based on synthesized participants’ voices about the awareness of DPP.

Constructed Reflections Based on a Synthesis of Participants’ Voices	Excerpts from Participants’ Transcripts
I became aware of disaster planning and preparedness because … *	The rationale for DPP awareness, including the reasons and methods that led the participants to be aware of their hospitals’ DPP documents and processes
	*“It is my job; I work in disasters”* (H1-6). *“My role is one of the first to be made aware of that”* (H2.1). *“My role description states I need to be aware of that”* (H2.6). *“I was a member of the … Committee, later, I became the Chair of the … committee. So that’s how I became aware, and because every time there’s a change to the doctrine within the emergency planning space it goes out to the committee, and I review it and provide feedback”* (H2.2). *“Disaster and emergency management training for the hospital executive and anyone else that would be identified as playing a key role in the Emergency Operations Center”* (H1.4). *“Those plans are all made available to all the staff, but they’re often not known by staff because the average staff member in a hospital … don’t need to know”* (H1.6).
My knowledge/competencies about DPP are….	The DPP awareness level, including the participants’ perception and evaluation of their or others’ knowledge and competencies a result of this awareness.
	*“At my level… I should know, at least my hospital’s disaster management plan”* (H1.1). *“I do not think I really became aware of them”* (H2.3). *“There was not a lot of disaster management, … there are some online modules around disaster management, I am not sure if they are mandatory though”* (H2.7). *“For my ward, would have a very basic knowledge”* (H1.1). *“People were very just unaware”* (H2.5). *“Their lack of knowledge or understanding can really impact the success of a response”* (H1.6). *“I don’t feel that that is effective …”* (H1.6). *“That was really about the lack of knowledge that the supply chain was damaged”* (H2.6).

* Column 2 quotations are reflections in completing the statements noted in Column 1.

**Table 2:** Disaster planning and preparedness (DPP) training: This is a table that shows excerpts from participants’ transcripts and the constructed reflections based on synthesized participants’ voices about DPP training.

Constructed Reflections Based on a Synthesis of Participants’ Voices	Excerpts from Participants’ Transcripts
I figured out that my training is … *	Quality of DPP training: including the participants’ perceptions, expectations and thoughts regarding their DPP training and practice.
	*“There is a minimum requirement that the Australian hospital must exercise its disaster management preparedness plans once a year, so that can be as simple as everybody checking their business continuity plan and practising what would happen if a pipe burst in a bathroom, and they had to close a ward that would technically meet the requirement, but it does not necessarily mean that it was good, effective training and knowledge sharing”* (H1.6). *“We do not expect nurses and doctors to pick up fire extinguishers unless they are comfortably trained, and it is appropriate to do so. So, we do not model a first responder, we model our safety first … We have never had the levelling of a facility”* (H2-6). *“For disaster education itself, there is still room to move but there are opportunities and people wish to take it up…basically, where are we always taught that if we cannot deal with it. It gets escalated higher …I think that is definitely something that pandemic planning has highlighted unless you’ve you have been in one a disaster you are quite naive to how it rolls out, and how that recovery happens afterwards”* (H1.2). *“It took a lot to get the information out to people and to understand people’s needs”* (H2-5).
What I need regarding training is …	Recommendations: including the participants’ recommendations and suggestions on their DPP training and in particular the simulations.
	*“We need to do a little bit more at a hospital level, not just emergency and ICU”* (H1.1). *“I believe that business continuity or disaster preparedness and planning or some kind of awareness of health emergency disaster and incident response should be included in that training”* (H1.6). *“Having some kind of awareness training and as a part of mandatory training or induction training would be really important and that’s updated regularly…. And the second thing that I think would be really valuable is to involve all levels of staff in training”* (H1.6). *“So do we need to look at upskilling our staff because we have a very junior team… we do have a high turnover of Emergency Nurses. So, looking at the capabilities that we had within our staff to work in different areas”* (H1.2). *“What we should be doing is continuing to work up those simulations”* (H2.6). *“I had to go back and look at the training, make the modifications to the training to make sure that everybody makes what they needed to do…What happens if electronics broke down. And we go through manually and get all these sorts of things done. So, incorporating that into future training as well”* (H2.4).
What I need regarding simulation is …	The significance of time: including the effect of time on the quality and application of DPP training,
	*“Simulations are like reading a textbook. … does not account for the actual human factor, and all the other factors that are involved in it”* (H1.1). *“Looking at the fight or flight type scenario of… and start trying to knock it down until emergency services come to apply what they learn”* (H2.4). *“We have got to make sure that what we do on what we train is going to be sustainable for the people that are going to be doing the actual responses in their work area, learn by doing, by doing very quickly…Apart from learning from past mistakes, would be to have more hands-on facilities available where staff could actually undergo physical training”* (H2.4). *“If we have got a fire in that building. How are we going to get these people out and run different scenarios through software? Love to get the software that fire engineers have because they can run all that sort of thing … that is a big financial thing and you’ve got to look at all the ins and outs and things like that”* (H2.4).

* Column 2 quotations are reflections in completing the statements noted in Column 1.

**Table 3:** The significance of time with regard to disaster planning and preparedness (DPP): This is a table that shows excerpts from participants’ transcripts and the constructed reflections based on synthesized participants’ voices about the significance of time with regard to DPP.

**Constructed Reflections Based on a Synthesis of Participants’ Voices**	**Excerpts from Participants’ Transcripts**
The time factor affected me by … *	The significance of time: including the effect of time on the quality and application of DPP training
	*“Preparedness is well written currently programs to provide evidence, not that anyone ever reads them…the amount of time that we put into it, but we have to have written to support our decision making …”* (H1.3). *“It hadn’t been put into place enough times … people were very just unaware and so it took a lot to get the information out to people and to understand people’s needs”* (H2.5). *“We spend a lot of time doing one on one education, and that’s exhausting …”* (H1.3). *“The slow response from staff could have made things a lot worse …”* (H2.4). *“We weren’t needing to be trained in the vaccination space that had to change very quickly and so that the agility was definitely there”* (H1.4).

* Column 2 quotations are reflections in completing the statements noted in Column 1.

**Table 4:** Wellness and needs: This is a table that shows excerpts from participants’ transcripts and the constructed reflections based on synthesized participants’ voices about their wellness and needs.

Constructed Reflections Based on a Synthesis of Participants’ Voices	Subtheme: Fear and Vulnerability
**My feelings and concerns are/were …**	*“We would expect that a nurse would want to come in and treat sick people, but we faced a new challenge where people did not want to do that, for fear that they would, therefore, go on and infect their families”* (H2.5). *“Whereas in floods and in fire and mass casualty our staff are very well prepared to enact that but the personal safety aspect and the wellbeing of their families and friends during COVID, was a response problem, not a problem but something we encountered that we had not kind of perceived was going to be as bigger concern”* (H2.5). *“Very senior doctors that literally could do mass casualties and save people’s lives so were refusing to come to work because they were scared* (GCG2-5). *I have actually been verbally abused multiple times by work colleagues, which is very distressing, and it is still ongoing with the vaccine, vaccinations stuff because it is fear and lack of understanding”* (H1.3). *“They are scared, and I respect and understand they are scared”* (H1.3). *“We have about 436 vulnerable employees registered, and there was a lot of deployment required for those people … Convincing staff that you know you’ve got to stay safe or otherwise you can’t safely treat patients” (H2.2).* *“When you are in a bit of a panic inside … you forget what you have also been taught as well”* (H1.2). *“External pressures on them from work from home …. They may have relatives who are sick, they cannot go overseas to see their family”* (H1.5). *“So anecdotally, on the ground, they are tired, they are stressed. They do not really know if they can plan their overseas holidays to see they are going relatives because they do not know if they can get there…. All of my staff of work the last five public holidays they do not get public holidays anymore. And they didn’t get any leave over Christmas. So also, you know they’re wearing this PPE; they’re wearing n95 masks, face shields all day, every day. It is a lot of physical toll on them also. So heaps of things that go into it”* (H1.5).
	**Sub-Theme: Doubts and Uncertainty**
	*“Facing a new kind of heightened concerns because people, you know, were not quite sure how the disease was transmitted, or chronic infection might occur, so there was a lot of concerns around”* (H2.5). *“You never know what’s around the corner”* (H1.-2). *“With COVID, it’s too much information that they’re not sure how to respond”* (H1.-5). *“If we do have a major incident, we can escalate it up one more one. There is no set criteria for what we call level one, level two or level three is purely based on the incident itself, what impact it’s going to have on people”* (H2.-4). *“Whilst we have plans in place for avian flu for Ebola, this is was not that moving faster, then changes move, and our ability was the second problem with things outside”* (H2.-6). *“COVID has had many changes in 18 months. So, I do the risk assessments; initially in 2020 were wonderful, but there has not been a lot of room to review them”* (H2.-7). *“The problem with disasters is people’s reaction to disasters. So generally, it’s a heightened state. And they are looking for guidance where necessary; they will not necessarily be there. So they just have to do the task that they are given. And then afterwards, have a debrief about it, but staff reactions to what they are asked to do, and disasters is a problem, and sometimes they just do not do it”* (H1.-5). *“I look and think, how will we cope?”* (H1.-1).
**My resilience and adaptation levels are …**	**Sub-Theme: Competing priorities**
	*“There was a flood, our staff can’t get to work, but our patients still can. So we may have patients that live inside the catchment but staff that travel a long way to get to work. So we still have to provide care for these patients, even though we may have reduced resources. So that was a huge problem that was faced”* (H1.-5). *“And we look at necessary work versus unnecessary work … we are going to need to be busier than we ever were to meet the targets that we need to have because we had months where we were not operating, and also waitlists are growing”* (H2.-5). *“I feel like they have a number of competing priorities, and between managing the pandemic and managing the staff and managing rosters and patients and all of that”* (H2.-7).
**My resilience and adaptation levels are …**	**Sub-Theme: Resilience, and Adaptation**
	*“This isn’t what they normally do. This isn’t in our job description. We haven’t had to do anything outside of that.... So, how do we adapt to that? So I think people’s ability to respond to change has decreased, as it has just kept going … There was at first COVID way the second COVID way, the bushfires the flood and I just think that they can’t, they don’t have that resilience in themselves anymore to continue to change because they don’t really know where they fit in … But I think the staff themselves are probably not as resilient as they once were”* (H1.-5).
**My needs to be considered are …**	**Sub-Theme: Needs Considerations**
	*“So it’s not just our capability, it is our equipment and everything and the department is looking at actual capabilities … Your mental state afterwards as well or during it. So I think there has been more focus on wellness for staff, especially with this pandemic. … looking at how we can help staff … through wellness through activities, and I think there’s been a huge push for that”* (H1.2). *“We just enhanced the plans that further because we went further into the psychological wellness of the employees as well”* (H2.-2). *“If we have a really big disaster, we need a lot of ventilators or things like that and, I think that is where the government would probably need to step in and would step in and say, well, we need to use these resources over here in the private sector”* (H1.1). *“As a health service had done disaster planning but had failed to consider the establishment of a well- being Committee to order a well-being oversight arm of the disaster response team to ensure that our staff were feeling supported during the initial stages of the pandemic and what I mean by that is that we treated them as health professionals were, as we had not quite grasped the need to treat them as both health professionals and people”* (H2.-5). *“It is just looking at our capabilities anticipating and responding to this, looking at how we should at least have a look at what we need to do to plan for preparedness, looking at this thing we are looking at as our staff preparedness, so how they are prepared for a disaster. And they are going to need and the resilience as we go forward and looking at the wellness as well”* (H1.2).

## Data Availability

The data supporting the results are in the form of interviews transcripts and can be made available by written request of the corresponding author.
